# Pharmacodynamics of Ceftiofur Selected by Genomic and Proteomic Approaches of *Streptococcus parauberis* Isolated from the Flounder, *Paralichthys olivaceus*

**DOI:** 10.1155/2020/4850290

**Published:** 2020-03-31

**Authors:** Naila Boby, Muhammad Aleem Abbas, Eon-Bee Lee, Seung-Chun Park

**Affiliations:** Laboratory of Veterinary Pharmacokinetics and Pharmacodynamics, College of Veterinary Medicine, Kyungpook National University, Daegu 41569, Republic of Korea

## Abstract

We employed an integrative strategy to present subtractive and comparative metabolic and genomic-based findings of therapeutic targets against *Streptococcus parauberis*. For the first time, we not only identified potential targets based on genomic and proteomic database analyses but also recommend a new antimicrobial drug for the treatment of olive flounder (*Paralichthys olivaceus*) infected with *S. parauberis*. To do that, 102 total annotated metabolic pathways of this bacterial strain were extracted from computational comparative metabolic and genomic databases. Six druggable proteins were identified from these metabolic pathways from the DrugBank database with their respective genes as mtnN, penA, pbp2, *murB*, *mur*A, coaA, and fni out of 112 essential nonhomologous proteins. Among these hits, 26 transmembrane proteins and 77 cytoplasmic proteins were extracted as potential vaccines and drug targets, respectively. From the FDA DrugBank, ceftiofur was selected to prevent antibiotic resistance as it inhibited our selected identified target. Florfenicol is used for treatment of *S. parauberis* infection in flounder and was chosen as a comparator drug. All tested strains of fish isolates with *S. parauberis* were susceptible to ceftiofur and florfenicol with minimum inhibitory concentrations (MIC) of 0.0039–1 *μ*g/mL and 0.5–8 *μ*g/mL, IC_50_ of 0.001–0.5 *μ*g/mL and 0.7–2.7 *μ*g/mL, and minimum biofilm eradication concentrations (MBEC) of 2–256 *μ*g/mL and 4–64 *μ*g/mL, respectively. Similar susceptibility profiles for ceftiofur and florfenicol were found, with ceftiofur observed as an effective and potent antimicrobial drug against both planktonic and biofilm-forming strains of the fish pathogen *Streptococcus parauberis*, and it can be applied in the aquaculture industry. Thus, our predictive approach not only showed novel therapeutic agents but also indicated that marketed drugs should also be tested for efficacy against newly identified targets of this important fish pathogen.

## 1. Introduction

*Streptococcus parauberis* is one of the major pathogenic bacteria which cause economic losses in the aquaculture industry in the Northeast Asia fish farming industry, including Korea. *Streptococcus parauberis*, a member of the *Streptococcaceae* family, is a nonmotile, Gram-positive, alpha-hemolytic lactic acid bacterium with a coccoid shape. It is closely related to *S. uberis* and is included in the pyogenic streptococci class. In freshwater and marine cultures, it was first found in *Scophthalmus maximus* (turbot) and is the leading cause of chronic streptococcal infection, but it was initially included in the *S. uberis* subtypes [1, 2]. Streptococcal diseases of aquaculture fish are some of the most disastrous pathogenic conditions worldwide, including different regions of Spain, the US, Korea, Japan, Israel, and Italy [3–7].

With a progressively developing aquaculture industry, bacterial pathogen infections have increased, causing exponential losses of different fish species based on their geographical regions like *Paralichthys olivaceus* (olive flounder) in South Korea and Japan, *Scophthalmus maximus* (turbot) in Spain, *Sebastes ventricosus* (sea bass species) in Japan, and *Morone saxatilis* (striped bass) in North America due to streptococcosis. It produces a considerable deficit economically to fish farmers because of its substantial impact on fish stock mortality, and its occurrence must be controlled [6, 8].

On the Korean peninsula, *Paralichthys olivaceus* (olive flounder) is one of the dominant marine and fresh water fish that has suffered consistent mortality. In every geographical region, its pathogen is unaffected and has developed resistance against many antibiotics [1, 2]. The resistance issues demand exploration of new alternative therapeutic targets for treatment of infection or finding those targets, which will enhance antimicrobial sensitivity that is already present.

With increasing knowledge regarding an infecting organism's genome, metabolism, proteomes, and molecules important for their survival, the discovery of new drug targets is easier than before. Moreover, the number of microbial species with completely sequenced genomes is increasing frequently. Currently, the number of reported prokaryotic whole genome sequences is greater than 2000. The use of computational proteomic, genomic, and bioinformatic analyses for investigation of such infectious pathogenic organisms has not only aided in the *de novo* discovery of efficacious therapeutic agents but also provided alternative uses of already available drugs [9, 10].

Various computational comparative genomic and bioinformatic analysis methodologies are established for discovery of potential therapeutic agents against many microbial agents using comparison of host and pathogen proteins [11]. Pathogenic organisms have unique essential proteins that are necessary for the survival of an organism that can be targeted for therapeutic applications for control of bacterial growth [12]. Moreover, the use of existing therapeutic agents specifically for newly identified targets will not only save drug development time but also reduce drug treatment costs.

Cephalosporins are one of the most important antibacterial classes, as four generations from this drug class have beta-lactamase resistance due to the presence of a beta-lactam ring in their substructure, similar to penicillin. Although a large number of human drugs belong to this class, their use in dairy cattle and the aquaculture industry is limited. For treatment of mastitis infections in dairy cattle, only a few cephalosporins belonging to the 1^st^ and 2^nd^ generation classes have been used globally under strict regulations. Later, 3^rd^ and 4^th^ generation drugs like ceftiofur and cefquinome, respectively, are administered for veterinary purposes [13].

Ceftiofur (sodium salt) is one of the novel 3^rd^ generation broad-spectrum cephalosporins introduced for veterinary use but is also administered for pathogenic bacterial infections in fish and poultry as a cell wall synthesis inhibitor. Ceftiofur is marketed under the brand name Naxcel® for Pasteurella infections of bovine respiratory disease treatment [14, 15]. Numerous reports showed that ceftiofur sodium controls infections in sheep, cattle, horse, swine, balady chicken, broiler chickens, and American black duck against *Pasteurella multocida*, *P. haemolytica*, and *E. coli*. In our study, we attempted to use ceftiofur sodium pharmacodynamics as an indicator of proof of our identified target by -omic study as a potential therapeutic target [16, 17].

Florfenicol is a semisynthetic antibacterial agent with a chemical structure and spectrum of antibacterial activity like thiamphenicol. Both florfenicol and thiamphenicol are chloramphenicol analogues in which the *p*-nitro group on the aromatic ring is substituted with a sulfonyl methyl group. Florfenicol binds to the bacterial 50S ribosomal subunit and inhibits protein synthesis at the peptidyl transferase step. *In vitro* investigations with florfenicol demonstrated potent activity against several bacteria pathogenic to fish. *In vivo* efficacy against furunculosis in Atlantic salmon and classical vibriosis in cod was confirmed [18].

Here, we identified putative novel therapeutic targets against *Streptococcus parauberis* by using available metabolic and genomic pathways and also recommended a new antimicrobial drug against *S*. *parauberis* infections in olive flounder with aid of the pharmacodynamics profiles of the approved antimicrobial drugs florfenicol and ceftiofur. We expect that our findings will not only give novel putative agents against *S. parauberis* but also provide new foundations using comparative and subtractive genomic methodologies for general pharmacodynamics studies of existing drugs or for specific organisms.

## 2. Materials and Methods

### 2.1. Metabolic Pathway Analysis

**Figure 1:**
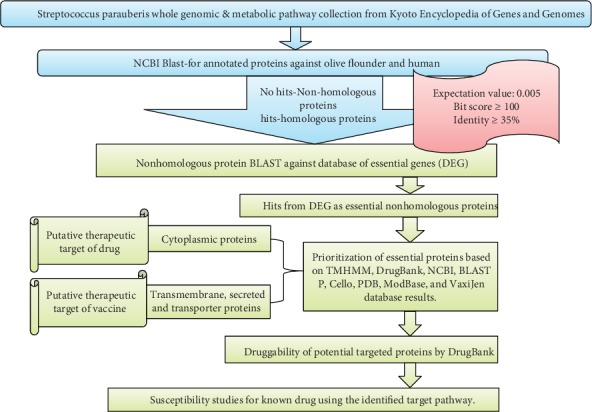
Illustration of the comparative and subtractive genomic target identification along with susceptibility studies in *Streptococcus parauberis*. Using the different databases at each step (KEGG, NCBI-Blast, and DEG), the essential hits were selected. The other databases (CELLO, ModBase, PDB, and VaxiJen) have been used to characterize the selected nonhomologous essential proteins for their 3D structure and other physical properties. Based on this characterization, the low molecular weight (<110 kDa) cytoplasmic proteins are considered putative drug targets whereas the transmembrane proteins are putative vaccine targets. Finally, the druggability of selected essential putative targets was analyzed using the DrugBank database. Afterward, one known antimicrobial drug was selected to target the *Streptococcus parauberis* strains and to check the susceptibility of planktonic and biofilm-forming bacteria. Schematic flowchart.

The KEGG database (abbreviated from the Kyoto Encyclopedia of Genes and Genomes) provides chemical, genomic, and functional information of the system of an organism and is used extensively as a source of reference dataset information produced by the sequencing of genomes [19]. In the present study, the metabolic pathways, protein, and nucleotide sequences of *Streptococcus parauberis* and *Homo sapiens* (human) genomes were retrieved from the KEGG pathway database [20, 21]. Different databases and search engines used during comparative genome analyses for the purpose of identifying therapeutic drug targets are demonstrated as a flowsheet in Figure 1.

### 2.2. Nonhomologous Essential Protein Screening

To identify essential nonhomologous proteins of the infecting bacteria, a comparison was performed in two steps. Firstly, we compared the proteins of *S. parauberis* with the host fish *Paralichthys olivaceus* (taxid: 8255) and with *Homo sapiens* (taxid: 9606) using NCBI-Blast for proteins with a threshold of 0.005 as an expectation value (*e* value), a percentage similarity of ≤35%, and a bit score of 100 as the minimum limit [22]. In the next step, these selected no-hits were used for comparison in the database of essential genes (DEG). The nonhomologous sequences of *S. parauberis* were aligned with the experimentally verified essential genes of five streptococcal species and proteins of 38 other Gram-positive and Gram-negative bacteria in the DEG-14.5 using the DEG microbial BLASTP with a minimum possible bit score of 100 and a cutoff *e* value of 10^−10^ [23, 24].

### 2.3. Characterization of Pathogen Nonhomologous Essential Proteins as Drug Targets

Based on their structural and molecular nature, the selected hits from the nucleotide and gene databases were characterized as therapeutic vaccine and drug targets. Proteins present in the cytoplasm are better drug targets; on the other hand, proteins present on the surface of cellular membranes are better targeted by vaccines [25]. Cellular localization analysis identifies proteins to their different locations on and within the pathogenic cell. The multiclass support vector machine classification database, also known as CELLO, version 2.5 (http://cello.life.nctu.edu.tw), was used for prediction of cellular and subcellular localization of the selected target proteins [26]. After cellular localization, the number of transmembrane helices in membrane proteins was predicted by TMHMM version 2.0 (http://www.cbs.dtu.dk/services/TMHMM/). This database is based on the hidden Markov model, and about 97–98% of the transmembrane helices have been determined by its experimentally predicted evidence [11, 27].

For determination of the molecular weight (MW) of the selected proteins, we used different online databases. As suggested by previous literature, smaller proteins are more soluble and easily purified, and such, they are more appropriate substances for development as drugs. Thus, we excluded proteins with MW > 110 kilodaltons [28].

Using these proteins for further analysis, the experimentally and computationally solved 3D structures were determined from the Protein Data Bank (PDB) (https://www.rcsb.org/pdb) and ModBase (https://salilab.org/modbase) databases, respectively [29, 30]. The PDB is a worldwide repository in which experimentally determined structures of proteins, nucleic acids, and complex biomolecule assemblies are deposited, and structures are explained by following their standards. ModBase is a database of protein structures developed by computational approaches and validated by statistically significant sequence alignments and model assessments [28].

In addition, for prediction of antigenic proteins, the database for protective antigen vaccine prediction, VaxiJen version 2.0, was used set with a threshold value > 0.4. The antigenic probability score, above the threshold value, represents the highest accuracy for the quantitative measure of protein sequences as protective antigens. These antigens form the basis of a subunit vaccine. A higher score of a protein refers to a higher probability for protective ability [31, 32].

The DrugBank database (version 4.3) contains unique bioinformatic and cheminformatic data of drugs and drug targets and is used for determining the druggability of essential proteins. By using default parameters, proteins were aligned for the available drug entries that included nutraceuticals, small molecule drugs, biotech (protein/peptide) drugs, and experimental drugs as approved by FDA (https://www.drugbank.ca/) [33].

### 2.4. *In Vitro* Efficacy Testing of Identified Protein Targets for Ceftiofur and Florfenicol

As ceftiofur acts as a penicillin-binding protein inhibitor, we obtained 21 strains of *S. parauberis* isolated from fish and analyzed the functional effectiveness of identified targets in these strains by ceftiofur, and florfenicol was used for comparative effects.

#### 2.4.1. Antimicrobial Susceptibility Profiles

*(1) Minimal Inhibition Concentration (MIC) and Minimal Bactericidal Concentration (MBC)*. The broth microdilution method was applied to determine the susceptibility of ceftiofur sodium and florfenicol (both obtained from Sigma-Aldrich, St. Louis, MO, US) against *S. parauberis* (21 isolates and 1 KCTC 3651 strain) and *S. aureus* ATCC 29213. Generally, the MICs of the isolates were determined according to the method by the Clinical and Laboratory Standards Institute (2017), in cation-adjusted Mueller-Hinton broth (Ca-MHB; BD Bacto™) using 5 × 10^8^ CFU/mL of bacterial concentrations [34]. The initial concentration of all antimicrobials was 1024 *μ*g/mL in Ca-MHB, and the stock solutions were filtered using a 0.22 *μ*m syringe filter (Merck Millipore Ltd., MA, US). The MIC_50_ values (at which 50% of the isolates were inhibited), MIC_90_ values, and MIC ranges of both antimicrobials against the isolates were determined. Bacteria were cultured overnight, adjusted with TH broth to an optical density of 0.1 at 600 nm (OD600), and aliquoted into 96-well plates. The antimicrobials were serially diluted twofold with MHB to give a concentration range of 512 *μ*g/mL in 100 *μ*L volumes. The growth and negative controls were prepared using Ca-MHB with and without bacteria, respectively. Microtiter plates were incubated at 30°C for 24 h. The MIC was determined as the lowest concentration at which noticeable growth was not observed. The MBC (the lowest concentration that showed a 99.9% or higher killing rate) was determined by culturing 20 *μ*L of the drug dilutions with samples from the 96-well plates on MHA plates and incubating overnight. The distribution of colonies on plates was inspected visually to assess the drug carryover effect. Studies were conducted in duplicate and were repeated at least twice on separate days.

*(2) Mutation Prevention Concentration (MPC)*. The MPC determination for ceftiofur and florfenicol was performed as described previously [35, 36]. In summary, the tested bacteria were cultured, and after 24 h incubation in MHB, the bacterial suspensions were centrifuged at 5000g for 20 min and resuspended in 3 mL MHB to yield a concentration of 10^10^ CFU/mL. By plating, the serial dilutions of 100 mL of samples on a drug-free medium inoculum were further confirmed. After, agar plates containing seven different known concentrations of ceftiofur and florfenicol were inoculated with aliquots of 100 *μ*L of *Streptococcus parauberis* strains (approx. 10^10^ CFU). *S. parauberis* KCTC 3651, the fully susceptible control strain, was used as a control in each experiment. The inoculated plates were incubated for 48 h at 30°C and screened visually for growth. The minimum antibiotic concentration with no bacterial colonies present was recorded as the MPC.

#### 2.4.2. Time-Kill Curve Assay

To determine the killing dynamics of ceftiofur and florfenicol, a time-kill assay according to the CLSI (NCCLS, 1999) guidelines was performed [37]. In summary, exponentially growing overnight cultures of tested *S. parauberis* strains were diluted to ∼5 × 10^8^ CFU/mL bacterial densities and then inoculated to 5 mL brain-heart infusion (BHI) medium tubes. Later, ceftiofur and florfenicol were added to these tubes after bacterial dilutions at concentrations of 0.5, 1, 2, and 4x MIC, whereas one tube with no antibiotic was used as the control for growth. After incubating the samples at 30°C on a shaking incubator, 20 *μ*L samples were taken from each tube at intervals of 0, 1, 2, 4, 6, 8, 12, and 24 h. Each sample was diluted 10-fold serially into aliquots and inoculated on BHI agar plates for the estimation of CFU per mL. For the two antibiotics, time-kill curves were obtained at different concentrations.

#### 2.4.3. Biofilm Formation Quantification

For quantifying biofilm formation of *Streptococcus parauberis*, we followed the method of O'Toole (2011) with some bacterial growth modifications [38]. In summary, using the BHI broth, we prepared 100x dilutions from the 24 h inoculum of nine tested strains for determination of bacterial attachment and biofilm formation. The dilutions were transferred into 96-well microtiter polystyrene plates (Nunclmmuno™ MaxiSorp™, Nalgene, US) as eight replicates. After 48 h incubation at 30°C, we measured optical density at the wavelength of 595 nm and estimated the bacterial growth for both adherent as well as suspended cells [39]. We discarded the supernatant and rinsed the wells 3–4 times with 400 *μ*L per rinse of distilled water. Following rinsing, wells were stained using 125 *μ*L volume of 1% crystal violet solution. After 20 min, the dye was discarded and wells were washed using distilled water 3–4 times. The dye was dissolved using 125 *μ*L of acetic acid (30% *v*/*v*), and we quantified the biofilm formation of tested strains by measuring OD595.

#### 2.4.4. Minimum Biofilm Eradication Concentration (MBEC)

For measurement of *S. parauberis* strain biofilm sensitivity to ceftiofur and florfenicol, the biofilms of the selected strains were grown using Calgary Biofilm Device (CBD; Innovotech, Calgary, Canada) microplates with slight modifications from the manufacturer's instructions. In summary, freshly grown overnight cultures of selected strains were diluted to 0.5 McFarland standard in broth, and 100 *μ*L was transferred to 96-well flat-bottom microplates except for the negative control. Biofilm growth plates were immersed with modified CBD pegs and incubated to allow formation of biofilms for 48 h at 30°C. After rinsing, these biofilms containing peg lids with autoclaved distilled water were transferred to the antibiotic challenge plates with 100 *μ*L of Ca-MHB with twofold dilutions of antibiotics in each well. After 24 h of incubation with antibiotics at 30°C, we repeated the same rinsing procedure and placed the peg lids into the biofilm recovery plate with Ca-MHB free of antibiotics. Biofilms were transferred from pegs to the wells by sonicating the recovery plates using a Branson 8200 sonicator (Emerson Electric Co., US) for 5 min at room temperature. Finally, the peg lids were replaced with standard lids, and the OD650 was recorded using a microplate colorimeter (VersaMax; Molecular Devices Corp., US). The lowest antibiotic dilution that caused inhibition of bacterial regrowth was recorded as the MBEC.

### 2.5. Statistical Analysis

The SAS statistical software version 9.4 (SAS Institute, Cary, NC, US) with one-way ANOVA was used for biofilm quantitation and growth of bacteria analysis, and Duncan's Multiple Range Test (DMRT) was used for evaluation of statistically significant differences among treatment groups, i.e., *p* < 0.05 was noted as statistically significant. Figure legends are used to designate statistics of various experiments.

## 3. Results

### 3.1. Comparative Genomic Analysis

For *Streptococcus parauberis*, 102 identified pathways were selected for analysis (Table 1). In comparison of the selected pathways of the pathogen with the existing 299 pathways of humans (*Homo sapiens*), 29 pathways were declared as unique to the pathogen. About 793 proteins are involved in these pathways, and out of these, only 277 were recognized as nonhomologues for both olive flounder and humans at the selected cutoff value of KEGG.

**Figure 2:**
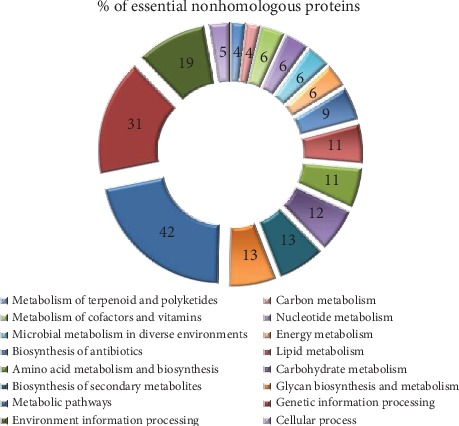
Distribution of essential nonhomologous proteins in major metabolic pathways of *S. parauberis*. Each color bar represents a single metabolic process. Essential nonhomologous protein distribution was analyzed using NCBI-Blast and the DEG database. The value noted in each bar represents the percentage of essential, nonhomologous proteins (out of 112 proteins as selected by DEG) involved in different metabolic pathways drawn manually from the KEGG pathway map.

In *S. parauberis*, 112 nonhomologous proteins were identified as essential when its nonhomologous proteins were aligned with the essential protein sequences of five different streptococcal species named as *Streptococcus agalactiae A909*, *Streptococcus pneumoniae*, *Streptococcus pyogenes MGAS5448*, *Streptococcus pyogenes NZ131*, and *Streptococcus sanguinis*. These 112 essential nonhomologous proteins take part in 16 major metabolic pathways noted from the KEGG database as shown in Table 2 and Figure 2.

**Figure 3:**
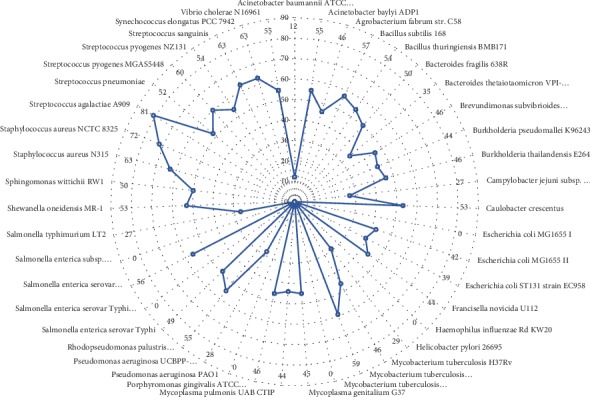
Frequency of hits of *S. parauberis* proteins by 43 bacteria in DEG. Distance from the center shows the extent of homologs of the 112 proteins by the essential proteins of 43 bacteria on DEG. Nonhomologous proteins of *S. parauberis* with homology were selected as essential and further characterized for selection of putative drug and vaccine targets.

Moreover, we compared all the essential proteins of the 43 available bacteria in the DEG with no selected hits. As described in Figure 3, the least hit was recorded by *Acinetobacter baumannii* ATCC 17978 with a homology of only 12 proteins, whereas the highest homology was recorded with the essential proteins of *Streptococcus agalactiae A909* with hits of 81 proteins. On the other hand, *Escherichia coli* strain MG1655 I, strain of *Mycobacterium tuberculosis* H37Rv III, *Salmonella enterica* Serovar *Typhi Ty Paoi*, *Pseudomonas aeruginosa* PAO1, *Haemophilus influenzae* RdKW20, *Salmonella enterica* subsp. *enterica serovar*, and *Typhimurium str.* 14028S each had no homology with the nonhomologous proteins of the pathogen under investigation.

The CELLO database analysis found the presence of 77 cytoplasmic, 24 membrane, and four extracellular proteins. Moreover, five proteins were cytoplasmic as well as membrane-bound and two proteins were found in three regions of the pathogen cell (cytoplasm, membrane, and extracellular area). Similarly, according to the TMHMM database, about 26 proteins had transmembrane helices but only six were in common with the CELLO database prediction ([Supplementary-material supplementary-material-1]). Furthermore, out of 26 transmembrane proteins, only 16 had an antigenic probability greater than 0.4 (Table 3).

Each essential protein's molecular weight was determined by referring to the UniProt database. As shown in Table 2 and [Supplementary-material supplementary-material-1], out of 112 nonhomologous essential proteins, only one protein showed a greater weight than the selected limit of molecular weight (<110 kDa) although all of them had 3D experimental models. The druggability results of the essential proteins as determined by the DrugBank database identified six proteins, which had hits for drugs that are approved, nutraceutical, investigational, and experimental at an *e* value limit of 10^−25^ (Table 4).

The six genes for these essential proteins with a hit from the DrugBank database were mtnN (5′-methylthioadenosine/S-adenosylhomocysteine nucleosidase), penA (penicillin-binding protein 2B), pbp2 (penicillin-binding protein 2), *murB* (penicillin-binding protein 1B), murA (penicillin-binding protein 1A), coaA (pantothenate kinase), and fni (isopentenyl-diphosphate delta-isomerase). The number of hits increased with an increase in expectation value, as 10 and 19 essential nonhomologous proteins were hits for expectation value limits of 10^−10^ and 10^−5^, respectively.

### 3.2. *In Vitro* Efficacy of Identified Proteins Targeted by Ceftiofur and Florfenicol

#### 3.2.1. *In Vitro* Antibiotic Sensitivity Profiles including Minimal Inhibition Concentration (MIC), Minimal Bactericidal Concentration (MBC), and Mutant Prevention Concentration (MPC)

The antimicrobial susceptibility profile against 22 field isolates from fish and a known strain (KCTC 3651) of *S. parauberis* was determined for the selected veterinary antimicrobial drugs. Ceftiofur sodium has an antimicrobial mechanism in common with the one of the identified therapeutic targets (PBP 2A) from the genomic analysis, and for comparative purposes, florfenicol was used.

**Figure 4:**
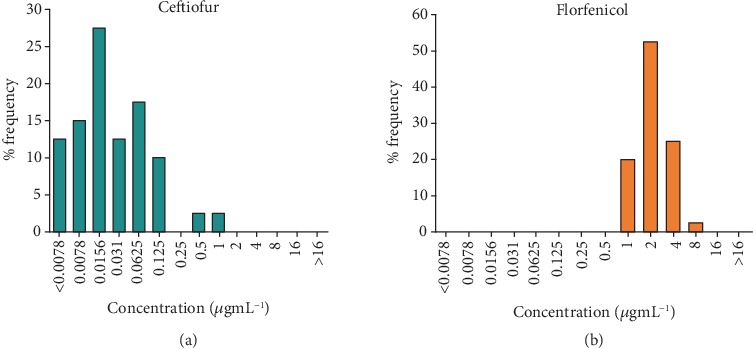
Minimum inhibitory concentration (MIC) frequencies. MIC frequencies observed for ceftiofur (a) and florfenicol (b) against 22 *Streptococcus parauberis* isolated strains from diseased olive flounder.

According to the MIC and MBC testing results, all isolates were completely sensitive to ceftiofur and florfenicol with MICs ranging from 0.0039 to 1 *μ*g/mL and 0.5 to 8 *μ*g/mL, respectively. The MBC values against field isolates ranged from 0.0078 to 32 *μ*g/mL and 1 to 128 *μ*g/mL for ceftiofur and florfenicol, respectively, as shown in Table 5 and Figure 4. The MPC for both antibiotics was evaluated in triplicate, and the MPCs of ceftiofur and florfenicol were more than the twofold dilution of their MIC. Hence, the MPC/MIC ratio for ceftiofur was 8–32, but in reference to florfenicol, this ratio was noted as eight (Table 6).

#### 3.2.2. Time-Kill Curve Assays and IC_50_ Values

**Figure 5:**
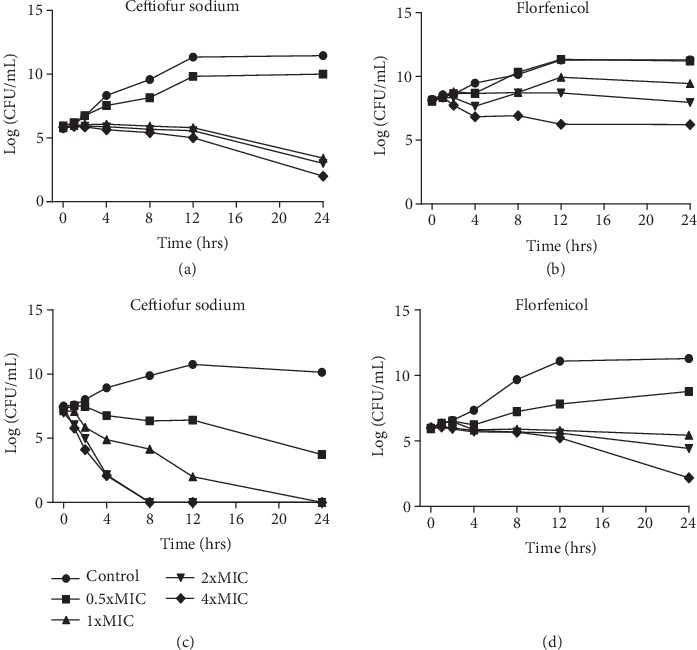
Time-kill curves of ceftiofur (a, c) and florfenicol (b, d) against *S. parauberis* KCTC 3651 strain (top) and *S. parauberis* S2628 strain (bottom). (a, c) represent inhibitory activities of ceftiofur at 0, 1, 2, 4, 8, 12, and 24 hours, and (b, d) represent the same for florfenicol when the drugs were exposed to exponentially growing *S. parauberis* KCTC 3651 and S2628 strains at their 0.5, 1, 2, and 4x minimum inhibitory concentration (MIC) values.

The inhibitory effects of ceftiofur and florfenicol were determined against *S. parauberis* using a time-kill assay by the growth inhibition method. The bacterial incubation was performed with each tested antibiotic at concentrations of 0.5, 1, 2, and 4x MIC. The killing dynamics of each antimicrobial showed that ceftiofur started to inhibit the tested strains after 4 hours, and at 12 hours, this inhibition was maximal. However, in the case of florfenicol, inhibitory activity also began after 4 hours, but maximum inhibition was noted at 24 hours. Moreover, the inhibition of bacterial growth by florfenicol was less than that of ceftiofur at 0.5x MIC (Figure 5).

By comparing the counts of bacteria after 12 hours of incubation using each antibiotic, the IC_50_ values were determined to obtain the growth inhibition concentrations of ceftiofur and florfenicol. The concentration of ceftiofur and florfenicol for 50% growth inhibition ranged from 0.001 to 0.5 *μ*g/mL and 0.7 to 2.7 *μ*g/mL, respectively (Table 5).

#### 3.2.3. Biofilm Quantitation and Minimum Biofilm Eradication Concentration (MBEC)

**Figure 6:**
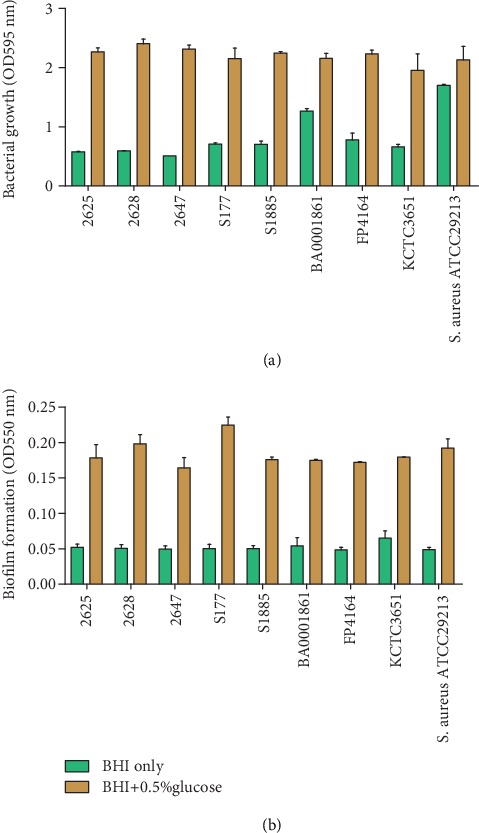
Quantitation of bacterial growth and biofilm formation in *Streptococcus parauberis* strains. Bacterial growth (a) vs. biofilm formation assay (b) of planktonic bacteria in brain heart infusion (BHI) and 0.5% glucose-supplemented BHI media. *S. aureus* is used as the positive control for biofilm formation whereas medium only is used as the normal control (NC). The optical density (OD) was read at wavelength of 550 nm. The absorbance was noted as <0.1 for weak biofilm formation, between 0.1 and 1 as moderate biofilm, and ≥1.0 as strong biofilm.

For quantitation of bacterial growth and biofilm formation activities of seven *S. parauberis* strains, BHI media and 0.5% glucose-aided BHI media were used. *S. aureus* ACTC 29213 was used as the positive control for biofilm formation (Figure 6). The growth of bacteria in BHI media ranged from OD values of 0.5 to 1.2. However, this range for growth of bacteria in 0.5% glucose-aided media was 2.1–2.4. For *S. aureus* ACTC 29213 at OD_550_, biofilm formation in 0.5% glucose-aided media was 0.1. Although the biofilm formation in BHI for other strains was <0.05, in glucose-aided BHI media, the formation of biofilm was moderate for all strains in the range of 0.1–0.2. If the absorbance was measured at <0.10 at OD_550_, the formation of biofilm was presumed as weak/absent, between 0.1 and 1.0, it was considered moderate, and if it was >1.0, it was determined as a strong biofilm.

The minimum biofilm eradication concentration (MBEC) was measured to check the ceftiofur and florfenicol susceptibility against biofilm-forming isolates of *S. parauberis* as shown in Table 7. Larger differences between the MBEC/MIC ratios show higher susceptibility differences of biofilm-forming bacteria versus planktonic bacteria towards ceftiofur and/or florfenicol.

## 4. Discussion

The increase in bacterial pathogenicity and resistance to antibiotics has provoked the interest of researchers for new studies of health and pathogenic bacterial species. Around the globe, scientists and researchers are paying more attention to novel therapeutic targets for preventing resistance to bacterial infection. The availability of vast computational parameters and -omic data has increased the identification of suitable therapeutic agents. Inhibition of essential proteins can stop bacterial growth, as they are important for survival of the bacteria. Systematic comparative analyses of *Streptococcus parauberis* found 112 potential vaccine and drug targets in the DrugBank, out of which six essential targets had druggability due to homology. We further characterized these targets using different databases to differentiate between potential drug and vaccine targets. The drug targets in question are involved in different cellular activities like metabolism of purines and pyrimidines, replication of DNA, ribosomal synthesis, ABC transporter pathway, and nucleotide metabolism.

Apart from proteins that could be targeted, we found several metabolic pathways involved in bacterial resistance against various antibiotics. Our targeted pathogen can resist the action of the antibiotic vancomycin (VCM). The vancomycin resistance pathway expression is induced by two component systems, i.e., VanS-VanR and D-Ala-D-Lac or D-Ala-D-Ser depsipeptides which can replace the D-Ala-D-Ala dipeptide, resulting in inhibition of vancomycin binding to pentadepsipeptides [D-Ser] or [D-Lac]. The variation in D-Ala-D-Lac and D-Ala-D-Ser indicates high and low levels of resistance to VCM, respectively. Peptidoglycan modifications lead to successive cell wall formation, and this may affect resistance in the present organism [40].

The pathway for cationic antimicrobial peptide (CAMP) resistance is also present. In host defense mechanisms, CAMPs play a pivotal role against microorganisms as a component of the innate immune response. In fact, CAMPs kill bacterial cells by deterioration of the integrity of bacterial inner and outer membranes. However, some bacteria have developed several resistance mechanisms like efflux pumps in membranes, external trapping mechanisms, substitution of anionic cell surface contents with cations, crosslinking and biosynthesis of cell surface components, and peptidase production of CAMPs. Similarly, *Streptococcus parauberis* can develop resistance against CAMPs [41, 42].

Strains of *Streptococcus parauberis* have previously shown resistance against different antimicrobial drugs such as tetracycline, oxytetracycline, and erythromycin [43, 44]. Because of the chance of antibiotic resistance, there is a need for new and alternative therapeutic targets against this pathogenic bacterium. In the past, the biology of microbial agents has limited the identification of new antimicrobial and vaccinating agents. However, advancement in proteomic and genomic knowledge has led to milestones in investigating new vaccines and for finding targets for effective therapeutic agents [10].

The essential nonhomologous proteins of *S. parauberis* were recognized as important potential targets, providing new perspectives on therapeutic targets in the pathogen pathways with both safety and specificity. Using different therapeutic and vaccine agents against those genomic and proteomic parts of the pathogenic bacteria that are essential for its reproduction is critical during potential drug design. These agents can interact or affect the normal replication of the targeted microorganism [45].

After a protein is established as essential, it is important to determine the protein characteristics and localization within the cell. This can assist in understanding its function and nature for therapeutic targeting either as a vaccine or as a target for the antimicrobial function [46, 47]. Therefore, the identified cytoplasmic proteins can be used for targeting by antimicrobial drugs whereas the suggested membrane proteins with transmembrane helices can act as selected toxins or surface-exposed proteins and targeted by vaccine production, and these vaccines can initiate the immune response mediated by antibodies [48, 49].

The low molecular weight and druggability of these essential proteins noted according to the DrugBank database assisted in filtering possible targets. In addition to these findings, some nutraceutical, experimental, investigational, and approved therapeutic agents were assessed against the binding potential of essential nonhomologous proteins of *S. parauberis* and proved that these proteins are druggable and can be used as potential therapeutic targets. Moreover, different combinations of these drugs may be used to treat streptococcal infections of aquaculture habitats.

In this study, we identified twenty-nine FDA-approved drugs with a hit from the essential proteins of *S. parauberis*, out of which five were veterinary-approved drugs. The six identified genes targeted by these drugs are reported to have vital roles in bacterial metabolism. As noted, the mtnN gene is related to the methylthioadenosine/S-adenosylhomocysteine (MTA/SAH) nucleosidase, and its importance in bacteria has been appreciated previously. By inclusive analysis of its various roles, it is an integral component of the activated methyl cycle, which recycles adenine and methionine through S-adenosylmethionine- (SAM-) mediated methylation reactions, and also produces the universal quorum-sensing signal, autoinducer-2 (AI-2) [50]. Furthermore, murA, murB, penA, and pbp2 genes produce penicillin-binding protein types 1A, 1B, 2B, and 2, respectively, and these proteins are membrane carboxypeptidases and transpeptidases. Peptidases are required for regulation of chain length, glycan subunit polymerization, and muropeptide cross-linkages [51]. The pantothenate kinase (coaA) gene is related to the CoA biosynthesis pathway in bacteria and mammals. Pantothenate kinase is a key regulator of biosynthesis and directs the intracellular concentration of CoA through feedback regulation by CoA and its thioesters [52]. The isopentenyl-diphosphate delta-isomerase (fni) gene is related to isoprenoid metabolism. Isoprenoids play an important role in all living organisms, in mammals as steroid hormones, in plants as carotenoids, and in bacteria as ubiquinones or menaquinones. Isoprenoids are synthesized by consecutive condensations of the five-carbon precursor isopentenyl diphosphate (IPP) to its isomer dimethyl-allyl diphosphate (DMAPP). Isopentenyl diphosphate delta isomerase catalyzes an essential reaction in the biosynthesis of isoprenoids by converting IPP to DMAPP [53].

As most of the identified therapeutic targets play a pivotal role in cellular metabolism, by developing new efficacious therapeutic agents in a synchronized way, we can potentially control the infections caused by *S. parauberis*. Moreover, by using the results of this study, we can make significant innovations in testing the efficacy of available antimicrobial drugs. Here, to check the therapeutic efficacy of one of the identified targets in this study, we used an approved veterinary drug, ceftiofur, which inhibits the transpeptidation step of peptidoglycan synthesis during the formation of cell walls by binding to penicillin-binding proteins. For comparative purposes, we also studied florfenicol [15].

The *in vitro* results for antibiotic sensitivity against the tested strains revealed the greatest effects of ceftiofur sodium against fish isolates of *S. parauberis* in comparison with florfenicol. All tested strains of *S. parauberis* showed 100% susceptibility to ceftiofur and florfenicol with an MIC range of 0.0039–1 *μ*g/mL and 0.5–8 *μ*g/mL, respectively. These results are common with other reported susceptibility studies of ceftiofur and florfenicol against streptococcal strains isolated from different sources [54–56].

Ceftiofur sodium and florfenicol have time-dependent inhibitory activities against *S. parauberis* strains, as bacterial growth was inhibited after 4 hours of incubation, irrespective of the antibiotic concentrations. Based on the minimum and maximum inhibitory concentrations, i.e., MIC_50_ and MIC_90_ (the concentration at which about 90 percent of the tested strains were inhibited), all tested bacterial strains were highly susceptible. We illustrated that ceftiofur has a minimum bactericidal concentration (MBC) 2–16 times greater than the MIC against *S. parauberis*, which confirmed reports by other authors [13, 16, 57, 58]. Although the IC_50_ range of ceftiofur was less than its MIC value, the IC_50_ value obtained from the time-kill curve analysis can assist in predicting the *in vivo* antimicrobial efficacy and dosage regimen according to the concentration change over time [59].

For environmental survival, one of the most important resistance mechanisms is the formation of biofilm by bacterial strains. Biofilms consist of aggregates of adherent bacteria in joint composition with proteins, polysaccharides, DNA, and lipids called an extracellular polymeric matrix. Different fish pathogenic bacteria including a few species of streptococcal bacteria like *S. mutants* have biofilm-forming potential [60–62].

Based on the present findings, the same characteristics of biofilm formation were identified within *S. parauberis* strains. Biofilm formation was enhanced with the addition of 0.5% glucose in BHI media as a source of carbohydrate. Biofilms work as the reservoir for survival of bacteria; thus, in this form, bacterial resistance to the antibiotics was increased. Due to the altered behavior of biofilm communities of bacteria, the antimicrobial susceptibility of biofilm-forming strains of *S. parauberis* was determined by the minimum biofilm eradication concentration (MBEC). The MBEC of ceftiofur was >1000 times higher than its MIC, which agrees with previous reports suggesting that the planktonic form of bacteria is 10–1000 times less resistant than bacteria of biofilm communities. In order to obtain maximum therapeutic outcomes and reduce antibiotic resistance, it is crucial to optimize the dosage of available antibiotics, and as such, agents for treatment of resistant bacterial infections are limited. Moreover, to achieve therapeutically effective antimicrobials, the application of biofilm-forming bacteria during *in vitro* susceptibility studies is more effective [63, 64].

In conclusion, our *in vitro* susceptibility studies found ceftiofur as an effective antibiotic against both planktonic as well as biofilm-forming strains of pathogenic *Streptococcus parauberis* isolated from fish. Moreover, we found that one of our identified target pathways was efficacious against all tested *S. parauberis* strains. In the future, more *in vitro* and *in vivo* findings will play a crucial role in validation of the present findings and in testing of other available antimicrobials against *S. parauberis*, for elucidation of efficacious and safe therapeutic agents. Furthermore, using available data for other identified pathways, we can develop more specific and potent novel therapeutic agents against *S. parauberis*.

## Figures and Tables

**Table 1:** Identified metabolic pathways of *Streptococcus parauberis* on Kyoto Encyclopedia of Genes and Genomes (KEGG).

KEGG ID^∗^	Pathways
stk00010	Glycolysis/gluconeogenesis—*Streptococcus parauberis*
stk00020	Citrate cycle (TCA cycle)—*Streptococcus parauberis*
stk00030	Pentose phosphate pathway—*Streptococcus parauberis*
stk00040	Pentose and glucuronate interconversions—*Streptococcus parauberis*
stk00051	Fructose and mannose metabolism—*Streptococcus parauberis*
stk00052	Galactose metabolism—*Streptococcus parauberis*
stk00053	Ascorbate and aldarate metabolism—*Streptococcus parauberis*
stk00061	Fatty acid biosynthesis—*Streptococcus parauberis*
stk00071	Fatty acid degradation—*Streptococcus parauberis*
stk00072	Synthesis and degradation of ketone bodies—*Streptococcus parauberis*
stk00130	Ubiquinone and other terpenoid-quinone biosynthesis—*Streptococcus parauberis*
stk00190	Oxidative phosphorylation—*Streptococcus parauberis*
stk00220	Arginine biosynthesis—*Streptococcus parauberis*
stk00230	Purine metabolism—*Streptococcus parauberis*
stk00240	Pyrimidine metabolism—*Streptococcus parauberis*
stk00250	Alanine, aspartate, and glutamate metabolism—*Streptococcus parauberis*
stk00260	Glycine, serine, and threonine metabolism—*Streptococcus parauberis*
stk00261	Monobactam biosynthesis—*Streptococcus parauberis*
stk00270	Cysteine and methionine metabolism—*Streptococcus parauberis*
stk00280	Valine, leucine, and isoleucine degradation—*Streptococcus parauberis*
stk00281	Geraniol degradation—*Streptococcus parauberis*
stk00290	Valine, leucine, and isoleucine biosynthesis—*Streptococcus parauberis*
stk00300	Lysine biosynthesis—*Streptococcus parauberis*
stk00310	Lysine degradation—*Streptococcus parauberis*
stk00330	Arginine and proline metabolism—*Streptococcus parauberis*
stk00332	Carbapenem biosynthesis—*Streptococcus parauberis*
stk00340	Histidine metabolism - *Streptococcus parauberis*
stk00350	Tyrosine metabolism - *Streptococcus parauberis*
stk00360	Phenylalanine metabolism - *Streptococcus parauberis*
stk00362	Benzoate degradation—*Streptococcus parauberis*
stk00380	Tryptophan metabolism—*Streptococcus parauberis*
stk00400	Phenylalanine, tyrosine, and tryptophan biosynthesis—*Streptococcus parauberis*
stk00430	Taurine and hypotaurine metabolism—*Streptococcus parauberis*
stk00440	Phosphonate and phosphinate metabolism—*Streptococcus parauberis*
stk00450	Selenocompound metabolism—*Streptococcus parauberis*
stk00460	Cyanoamino acid metabolism—*Streptococcus parauberis*
stk00471	D-Glutamine and D-glutamate metabolism—*Streptococcus parauberis*
stk00473	D-Alanine metabolism—*Streptococcus parauberis*
stk00480	Glutathione metabolism—*Streptococcus parauberis*
stk00500	Starch and sucrose metabolism—*Streptococcus parauberis*
stk00511	Other glycan degradation—*Streptococcus parauberis*
stk00520	Amino sugar and nucleotide sugar metabolism—*Streptococcus parauberis*
stk00521	Streptomycin biosynthesis—*Streptococcus parauberis*
stk00523	Polyketide sugar unit biosynthesis—*Streptococcus parauberis*
stk00525	Acarbose and validamycin biosynthesis—*Streptococcus parauberis*
stk00550	Peptidoglycan biosynthesis—*Streptococcus parauberis*
stk00561	Glycerolipid metabolism—*Streptococcus parauberis*
stk00562	Inositol phosphate metabolism—*Streptococcus parauberis*
stk00564	Glycerophospholipid metabolism—*Streptococcus parauberis*
stk00590	Arachidonic acid metabolism—*Streptococcus parauberis*
stk00592	Alpha-linolenic acid metabolism—*Streptococcus parauberis*
stk00620	Pyruvate metabolism—*Streptococcus parauberis*
stk00622	Xylene degradation—*Streptococcus parauberis*
stk00625	Chloroalkane and chloroalkene degradation—*Streptococcus parauberis*
stk00626	Naphthalene degradation—*Streptococcus parauberis*
stk00627	Amino benzoate degradation—*Streptococcus parauberis*
stk00630	Glyoxylate and dicarboxylate metabolism—*Streptococcus parauberis*
stk00640	Propanoate metabolism—*Streptococcus parauberis*
stk00643	Styrene degradation—*Streptococcus parauberis*
stk00650	Butanoate metabolism—*Streptococcus parauberis*
stk00660	C5-Branched dibasic acid metabolism—*Streptococcus parauberis*
stk00670	One carbon pool by folate—*Streptococcus parauberis*
stk00680	Methane metabolism—*Streptococcus parauberis*
stk00730	Thiamine metabolism—*Streptococcus parauberis*
stk00740	Riboflavin metabolism—*Streptococcus parauberis*
stk00750	Vitamin B6 metabolism—*Streptococcus parauberis*
stk00760	Nicotinate and nicotinamide metabolism—*Streptococcus parauberis*
stk00770	Pantothenate and CoA biosynthesis—*Streptococcus parauberis*
stk00780	Biotin metabolism—*Streptococcus parauberis*
stk00790	Folate biosynthesis—*Streptococcus parauberis*
stk00900	Terpenoid backbone biosynthesis—*Streptococcus parauberis*
stk00910	Nitrogen metabolism—*Streptococcus parauberis*
stk00920	Sulfur metabolism—*Streptococcus parauberis*
stk00970	Aminoacyl-tRNA biosynthesis—*Streptococcus parauberis*
stk01040	Biosynthesis of unsaturated fatty acids—*Streptococcus parauberis*
stk01100	Metabolic pathways—*Streptococcus parauberis*
stk01110	Biosynthesis of secondary metabolites—*Streptococcus parauberis*
stk01120	Microbial metabolism in diverse environments—*Streptococcus parauberis*
stk01130	Biosynthesis of antibiotics—*Streptococcus parauberis*
stk01200	Carbon metabolism—*Streptococcus parauberis*
stk01210	2-Oxocarboxylic acid metabolism—*Streptococcus parauberis*
stk01212	Fatty acid metabolism—*Streptococcus parauberis*
stk01220	Degradation of aromatic compounds—*Streptococcus parauberis*
stk01230	Biosynthesis of amino acids—*Streptococcus parauberis*
stk01501	Beta-lactam resistance—*Streptococcus parauberis*
stk01502	Vancomycin resistance—*Streptococcus parauberis*
stk01503	Cationic antimicrobial peptide (CAMP) resistance—*Streptococcus parauberis*
stk02010	ABC transporters—*Streptococcus parauberis*
stk02020	Two-component system—*Streptococcus parauberis*
stk02024	Quorum sensing—*Streptococcus parauberis*
stk02060	Phosphotransferase system (PTS)—*Streptococcus parauberis*
stk03010	Ribosome—*Streptococcus parauberis*
stk03018	RNA degradation—*Streptococcus parauberis*
stk03020	RNA polymerase—*Streptococcus parauberis*
stk03030	DNA replication—*Streptococcus parauberis*
stk03060	Protein export—*Streptococcus parauberis*
stk03070	Bacterial secretion system—*Streptococcus parauberis*
stk03410	Base excision repair—*Streptococcus parauberis*
stk03420	Nucleotide excision repair—*Streptococcus parauberis*
stk03430	Mismatch repair—*Streptococcus parauberis*
stk03440	Homologous recombination—*Streptococcus parauberis*
stk04122	Sulfur relay system—*Streptococcus parauberis*

^∗^KEGG ID represents the molecular pathways within major metabolic pathways such as cellular processes, genetic information processes, metabolism, and drug and disease development. Each pathway has a three-letter organism code (“skt” for *Streptococcus parauberis* in KEGG database) followed by a five-digit number.

**Table 2:** Distribution of essential nonhomologous proteins in major metabolic pathways of *S. parauberis*.

Major metabolic pathway	% of essential nonhomologous proteins^∗^
Metabolism of terpenoid and polyketides	4
Carbon metabolism	4
Cellular process	5
Metabolism of cofactors and vitamins	6
Nucleotide metabolism	6
Microbial metabolism in diverse environments	6
Energy metabolism	6
Biosynthesis of antibiotics	9
Lipid metabolism	11
Amino acid metabolism and biosynthesis	11
Carbohydrate metabolism	12
Biosynthesis of secondary metabolites	13
Glycan biosynthesis and metabolism	13
Environment information processing	19
Genetic information processing	31
Metabolic pathways	42

^∗^Essential nonhomologous proteins of *S. parauberis* selected by NCBI-Blast and DEG database analyses.

**Table 3:** *S. parauberis* nonhomologous essential proteins as subunits for vaccines with the number of transmembrane helices and antigenic characteristics predicted by TMHMM and VaxiJen databases, respectively.

Seq. KEGG ID	Genes	TMHMM^∗^	Probable antigenicity^∗∗^
STP_0829	mtlA	8	Antigen
STP_0495	atpB	5	Antigen
STP_0496	atpF	1	Antigen
STP_0274	pbpX	1	Antigen
STP_0314	dgkA	3	Antigen
STP_1416	bacA	8	Antigen
STP_1616	pbp1B	1	Antigen
STP_1749	pbp2A	1	Antigen
STP_0544	ltaS	5	Antigen
STP_1654	dppC	5	Antigen
STP_0122	lplB	4	Antigen
STP_0799	pstA	4	Antigen
STP_1210	lplB	6	Antigen
STP_1444	ecfT	4	Antigen
STP_0327	secG	2	Antigen
STP_1690	yidC, spoIIIJ, OXA1, ccfA	5	Antigen

^∗^Number of transmembrane helices for listed membrane proteins predicted by TMHMM (version 2.0) database. ^∗∗^Probable antigenicity (protective antigens and vaccine subunits) predicted by VaxiJen database (version 2.0) with threshold value 0.4.

**Table 4:** *S. parauberis* nonhomologous essential proteins with druggability for FDA-approved drugs as inferred from the DrugBank database using BLASTP and the list of FDA-approved drugs for the targets.

KEGG	Name	Gene	DB^∗^ ID	Drug name	Drug group
STP_0292	5′-Methylthioadenosine/S-adenosylhomocysteine nucleosidase	mtnN	DB02158	(1s)-1-(9-Deazaadenin-9-Yl)-1,4,5-trideoxy-1,4-imino-5-methylthio-D-ribitol	E
			DB02281	Formycin	E
			DB00173	Adenine	A, N
			DB02933	5′-Deoxy-5′-(methylthio)-tubercidin	E
			DB08606	(3R,4S)-1-[(4-Amino-5H-pyrrolo[3,2-D]pyrimidin-7-YL) methyl]-4-[(methylsulfanyl) methyl] pyrrolidin-3-OL	E

STP_1093	Penicillin-binding protein 2B	penA	DB01066	Cefditoren	A
			DB01212	Ceftriaxone	A
			DB01140	Cefadroxil	A, VA, W
			DB00493	Cefotaxime	A
			DB00319	Piperacillin	A
			DB00607	Nafcillin	A
			DB00415	Ampicillin	A, VA
			DB00485	Dicloxacillin	A, VA
			DB01163	Amdinocillin	W
			DB01603	Meticillin	A
			DB00456	Cefalotin	A, VA
			DB00713	Oxacillin	A
			DB01331	Cefoxitin	A
			DB00567	Cephalexin	A, VA
			DB03313	Cephalosporin C	E
			DB08795	Azidocillin	A
			DB00739	Hetacillin	A, VA, W

STP_1616	Penicillin-binding protein 2	pbp2	DB04147	Lauryl dimethylamine-N-oxide	E

STP_1749	Penicillin-binding protein 2	pbp2	DB04147	Lauryl dimethylamine-N-oxide	E
	Penicillin-binding protein 1B	mrcB	DB01598	Imipenem	A
			DB01329	Cefoperazone	A
			DB01332	Ceftizoxime	A
			DB01327	Cefazolin	A
			DB01331	Cefoxitin	A
			DB01328	Cefonicid	A
			DB01415	Ceftibuten	A
			DB00430	Cefpiramide	A
			DB00438	Ceftazidime	A
			DB00274	Cefmetazole	A
			DB00303	Ertapenem	A, I
			DB01414	Cefacetrile	A
			DB04570	Latamoxef	A
			DB06211	Doripenem	A, I
			DB11367	Cefroxadine	W
	Penicillin-binding protein 1A	mrcA	DB01598	Imipenem	A
			DB01329	Cefoperazone	A
			DB01332	Ceftizoxime	A
			DB01333	Cefradine	A
			DB01327	Cefazolin	A
			DB01331	Cefoxitin	A
			DB01328	Cefonicid	A
			DB01415	Ceftibuten	A
			DB00430	Cefpiramide	A
			DB00438	Ceftazidime	A
			DB00274	Cefmetazole	A
			DB00303	Ertapenem	A, I
			DB01414	Cefacetrile	A
			DB04570	Latamoxef	A
			DB06211	Doripenem	A, I

STP_0791	Pantothenate kinase	coaA	DB01783	Pantothenic acid	N, VA
			DB01992	Coenzyme A	N
			DB04395	Phosphoaminophosphonic acid-adenylate ester	E

STP_0603	Isopentenyl-diphosphate delta-isomerase	fni	DB03247	Riboflavin monophosphate	E

^∗^DB: DrugBank database; E: experimental, A: approved; VA: veterinary approved; W: withdrawn.

**Table 5:** Minimum inhibitory concentrations of ceftiofur and florfenicol against olive flounder isolated *Streptococcus parauberis*.

Parameters	Antimicrobial drugs	
	Ceftiofur	Florfenicol
MIC_50_ (*μ*g/mL)	0.0156	2
MIC_90_ (*μ*g/mL)	0.125	8
MIC_Range_ (*μ*g/mL)	0.0039-1	0.5-8
MBC_Range_ (*μ*g/mL)	0.0078-32	1-128
IC_50 Range_ (*μ*g/mL)	0.001-0.5	0.7-2.7
*R* (%)	0	0
KCTC 3651 (*μ*g/mL)	0.0078	0.5
*S. aureus* ATCC 29213 (*μ*g/mL)	1	2
CLSI range for *Streptococcus viridans*		
S	≤1	≤4
R	≥4	≥16
CLSI range for *Staphylococcus sp.*		
S	≤1	≤8
R	≥4	≥32

MIC: minimum inhibitory concentration; MBC: minimum bactericidal concentration; R: rate of resistance; CLSI range: clinical breakpoints for *Streptococcus* and *Staphylococcus* sp. as defined by the Clinical and Laboratory Standards Institute.

**Table 6:** Minimum inhibitory concentration and mutant prevention concentration comparison for ceftiofur and florfenicol against field and known strains of *S. parauberis*.

Strains	Ceftiofur			Florfenicol		
	MIC (*μ*g/mL)	MPC (*μ*g/mL)	MPC/MIC	MIC (*μ*g/mL)	MPC (*μ*g/mL)	MPC/MIC
*S. parauberis* 2628	1	32	32	2	16	8
*S. parauberis* KCCM3651	0.0078	0.0624	8	0.5	4	8

MIC: minimum inhibitory concentration; MPC: mutant prevention concentration.

**Table 7:** Minimum inhibitory concentration and minimum biofilm eradication concentration comparison for ceftiofur and florfenicol against field and known strains of *S. parauberis*.

Strain	Ceftiofur			Florfenicol		
	MIC (*μ*g/mL)	MBEC^∗^ (*μ*g/mL)	MBEC/MIC ratio	MIC (*μ*g/mL)	MBEC (*μ*g/mL)	MBEC/MIC ratio
*S. parauberis* 2628	1	4	4	2	8	4
*S. parauberis* S177	0.125	256	2048	4	16	4
*S. parauberis* S1885	0.0039	2	512	1	64	64
*S. parauberis* KCTC 3651	0.0078	32	4102	0.5	64	128
*S. aureus* ATCC 29213	1	256	256	2	64	32

MIC: minimum inhibitory concentration; MBEC: minimum biofilm eradication concentration.

## Data Availability

The data used to support the findings of this study are available from the corresponding author upon request.

## Supplementary Materials

Supplementary Materials Table S1: prioritization summary of 112 essential nonhomologous proteins of S. parauberis based on TMHMM, BLASTP, CELLO, PDB, and ModBase database results.
