# Supplementary material for: Pharmacodynamics of Ceftiofur Selected by Genomic and Proteomic Approaches of Streptococcus parauberis Isolated from the Flounder, Paralichthys olivaceus
Source: Int J Genomics. 2020 Mar 31;2020:4850290. doi: 10.1155/2020/4850290 (PMC7150728; doi:10.1155/2020/4850290)
Supplement: Supplementary Materials — Table S1: prioritization summary of 112 essential nonhomologous proteins of S. parauberis based on TMHMM, BLASTP, CELLO, PDB, and ModBase database results. [file 4850290.f1.pdf]

Table S1. Prioritization summary of 112 essential non-homologous proteins of *S. parauberis* based on TMHMM, BLASTP, Cello, PDB and Modbase database results.

SeqID	Protein ID	UNIPROT protein name	Cellular localization (CELLO)	TMHMM	3D Exp.	3D Model	Mol. Wt (Da)	Length
STP_1424	F6DF56	Fructose-1,6 bisphosphate aldolase, class II	C	No	yes	yes	32,988	305
STP_1660	Q9LLY5	Phosphoglycerate kinase	C	No	yes	yes	47,651	449
STP_0826	Q6QWF2	Mannitol-1-phosphate 5-dehydrogenase	C	No	yes	yes	40,577	375
STP_0829	Q9XBM7	PTS system mannitol-specific EIICBA component	M	8	yes	yes	67,698	635
STP_0125	P40714	Sucrose-6-phosphate hydrolase	C	No	yes	yes	54,363	477
STP_0159	Q74HI7	Invertase	C	No	yes	yes	55,769	489
STP_0202	A0A076Z4X0	Enoyl-CoA hydratase	C	No	yes	yes	28,732	263

STP_0204	Q5WEX8	3-oxoacyl-[acyl-carrier-protein] synthase 3	M	No	yes	yes	33,471	311
STP_0210	Q6QIW2	Biotin carboxyl carrier protein	C	No	yes	yes	27,603	260
STP_0211	A0A0D8ZBY3	3-hydroxyacyl-[acyl-carrier-protein] dehydratase FabZ	M, C	No	yes	yes	16978	150
STP_0213	C6VQ34	Acetyl-coenzyme A carboxylase carboxyl transferase subunit beta 2	M, C	No	yes	yes	30,944	281
STP_0214	A0A075MW59	Acetyl-coenzyme A carboxylase carboxyl transferase subunit alpha	C	No	yes	yes	28,629	257
STP_0942	A0A0E1EU54	Acyl-ACP thioesterase	C	No	yes	yes	29,202	250
STP_0494	A0A0C6FV48	ATP synthase subunit c	M	2	yes	yes	7,054	65
STP_0495	B5XKP6.1	ATP synthase subunit a	M	5	yes	yes	26,924	238
STP_0496	A0A017I3Q5	ATP synthase gamma chain	C	1	yes	yes	31,577	287

STP_0497	A2RFC5.1	ATP synthase subunit delta	C	No	yes	yes	19,917	178
STP_1339	Q9RRB7.1	Probable manganese-dependent inorganic pyro-phosphatase	C	No	yes	yes	34,039	314
STP_0002	Q9EVR1	DNA polymerase III subunit beta	M	No	yes	yes	41,867	378
STP_0074	Q6B8X4	DNA-directed RNA polymerase subunit alpha	C	No	yes	yes	35,640	314
STP_1058	P47473.1	Ribonucleoside-diphosphate reductase subunit alpha	E, M, C	No	yes	yes	82,249	721
STP_1079	Q8GN78	Hypothetical DNA polymerase III delta subunit	C	No	yes	yes	39,429	344
STP_1320	Q73GW0	DNA polymerase III, subunit, putative	C	No	yes	yes	31,175	273
STP_0304	Q5FJM5	Uridylate kinase	C	No	yes	yes	25,832	241
STP_1703	Q6Q8K8	Polyprotein	C	No	yes	yes	23,935	216

STP_0589	Q5X090	4-hydroxy-tetrahydrodipicolinate reductase	C	No	yes	yes	26,507	243
STP_0292	D4FIY6	5'-methylthioadenosine/S-adenosylhomocysteine nucleosidase	M	No	yes	yes	24,944	228
STP_0293	D4FIY6	5'-methylthioadenosine/S-adenosylhomocysteine nucleosidase	C	No	yes	yes	24,944	228
STP_1048	Q7N3N1	Uncharacterized protein	M, C	No	yes	yes	18,147	165
STP_1718	A0A0E2ETW6	2,3,4,5-tetrahydropyridine-2,6-dicarboxylate N-acetyltransferase	M, C	No	yes	yes	24,107	232
STP_1129	Q819Q2	UDP-N-acetylmuramoylalanine--D-glutamate ligase	C	No	yes	yes	48,954	450
STP_1373	A8Z4G7.1	UDP-N-acetylmuramate--L-alanine ligase	C	No	yes	yes	49,188	437

STP_0489	Q8XP97	Glucose-1-phosphate adenylyltransferase	C	No	yes	yes	43,889	393
STP_0501	Q989E5	UDP-N-acetyl glucosamine 1-carboxyvinyltransferase 1	C	No	yes	yes	45,535	430
STP_0633	Q5FUJ3	UDP-N-acetylenolpyruvoylglucosamine reductase	C	No	yes	yes	32,468	306
STP_1044	Q9S5W5	UDP-N-acetylglucosamine 1-carboxyvinyltransferase	C	No	yes	yes	44,819	419
STP_0972	A0A084AAF4	dTDP-4-keto-6-deoxyglucose-3,5-epimerase	C	No	yes	yes	22,359	197
STP_0973	Q7MPR1	Glucose-1-phosphate thymidylyltransferase	C	No	yes	yes	32,555	295
STP_0096	Q5ZFT1	MurM protein	M	No	yes	yes	47,436	407
STP_0274	Q54474	Penicillin-binding protein	M	1	yes	yes	72,907	666
STP_0314	A0A0D5N3T0	Diacylglycerol kinase	M	3	yes	yes	28,050	267

STP_0414	Q5ZFT1	MurM protein	C	No	yes	yes	47,436	407
STP_1093	Q841S2	Penicillin-binding protein 2B	E	No	yes	yes	21,032	199
STP_1332	O26630	UDP-N-acetylmuramyl tripeptide synthetase related protein	C	No	yes	yes	73,796	682
STP_1416	A0A014NGJ4	Undecaprenyl-diphosphatase	M	8	yes	yes	29,483	272
STP_1616	Q5ZGA3	Penicillin-binding protein 1b	E, M	1	yes	yes	49,137	443
STP_1749	Q83YQ8	Penicillin-binding protein 2A	E	1	yes	yes	80,806	740
STP_0022	A0A0H3LZN1	Phosphate acyltransferase	C	No	yes	yes	36,431	341
STP_0344	A0A0C6G3G2	Glycosyl transferases group 1 family protein	C	No	yes	yes	49,891	444
STP_0544	Q63324	Minimal change nephritis transmembrane glycoprotein	M	5	yes	yes	42,005	367

STP_0881	C0LGU8	LRR receptor-like serine/threonine-protein kinase FLS2	M	5	yes	yes	128,824	1,173
STP_1082	A0A0E1EMM8	Acyl-phosphate glycerol 3-phosphate acyltransferase	M	1	yes	yes	28,215	246
STP_0665	Q5WSY8	NAD kinase	C	No	yes	yes	33,088	295
STP_1255	A0A0H0XUT4	NH(3)-dependent NAD(+) synthetase	C	No	yes	yes	30,049	276
STP_1398	A0A0E2EG03	Probable nicotinate-nucleotide adenylyltransferase	C	No	yes	yes	24,441	210
STP_0779	A0A0E1EN83	Phosphopantothenate--cysteine ligase	C	No	yes	yes	25,743	230
STP_0791	Q1J6D9.1	Pantothenate kinase	C	No	yes	yes	35,610	306
STP_0632	P43777.1	2-amino-4-hydroxy-6-hydroxymethyldihydropteridine pyrophosphokinase	C	No	yes	yes	18,299	160
STP_0602	A0A0H2ZR70	Phosphomevalonate kinase	C	No	yes	yes	37,026	335

STP_0603	B8ZLF5.1	Isopentenyl-diphosphate delta-isomerase	C	No	yes	yes	37,687	336
STP_1701	Q8DVY1	Carbonic anhydrase	C	No	yes	yes	18,227	166
STP_0186	P63488	Glutamyl-tRNA(Gln) amidotransferase subunit A	C	No	yes	yes	52,801	485
STP_0506	Q836J5.1	Phenylalanine--tRNA ligase beta subunit	C	No	yes	yes	88,609	807
STP_1862	Q48QK9	Tryptophan--tRNA ligase	C	No	yes	yes	39,795	351
STP_1576	A0A0E1EEW9	Single-stranded DNA-binding protein	C	No	yes	yes	14,775	131
STP_1795	Q9KDI9	Holliday junction ATP-dependent DNA helicase RuvA	C	No	yes	yes	23,181	203
STP_1845	P25745	tRNA-specific 2-thiouridylase MnmA	C	No	yes	yes	40,959	368

STP_1654	Q5SHU8	Peptide ABC transporter, permease protein	M	5	yes	yes	43,616	395
STP_0122	Q9KWT8	AlgM1(Submitted name)	M	4	yes	yes	36,607	324
STP_0353	P73544	Glutamine-binding periplasmic protein/ glutamine transport system permease protein	M	3	yes	yes	57,681	530
STP_0354	Q5FMR1	Putative glutamine ABC transporter	M	5	yes	yes	59,725	537
STP_0438	P76344.1	Metal-binding protein ZinT	E, M	No	yes	yes	24,762	216
STP_0697	A0A0E1EVJ3	Adhesion protein	C	No	yes	yes	34,154	306
STP_0799	P31547	D-methionine transport system permease protein MetI	C	4	yes	yes	23,256	217
STP_0800	Q8A851	Phosphate transport system permease protein	C	6	yes	yes	43,507	398
STP_0884	Q8XIM2	Probable amino acid ABC transporter	C	4	yes	yes	55,706	502

STP_0885	P42400.2	Probable ABC transporter extracellular-binding protein YckB	M	No	yes	yes	31,720	287
STP_1210	Q9KWT8	AlgM1(submitted name)	M	6	yes	yes	36,607	324
STP_1444	Q03PY6	Energy-coupling factor transporter ATP-binding protein EcfA2	M	4	yes	yes	32,144	290
STP_0371	Q38ZK1	Two-component system, response regulator	C	No	yes	yes	27,312	237
STP_0793	Q93MY3	Sensor protein CovS	M	1	yes	yes	51,785	450
STP_0794	Q6AH14	Two-component system, regulatory protein	C	No	yes	yes	30,272	271
STP_1235	Q82BT0	Putative two-component system response regulato	C	No	yes	yes	25,891	239
STP_1428	Q630G2	Response regulator	C	No	yes	yes	28,433	246
STP_0242	A0A0H2UP84	Putative PTS system IIA component	C	No	yes	yes	18,872	165
STP_1054	Q7VP72	Phosphoenolpyruvate-protein phosphotransferase	C	No	yes	yes	63,240	573

STP_0050	Q8R7V6	50S ribosomal protein L23	C	No	yes	yes	11,165	96
STP_0053	Q661D7	50S ribosomal protein L22	C	No	yes	yes	15,217	133
STP_0056	B8H4E2	50S ribosomal protein L29	C	No	yes	yes	7,134	63
STP_0057	Q87E73	30S ribosomal protein S17	C	No	yes	yes	10,204	89
STP_0060	Q605C4	50S ribosomal protein L5	C	No	yes	yes	20,236	179
STP_0062	Q9G888	Ribosomal protein S8	C	No	yes	yes	14,929	130
STP_0064	Q9PJM9	50S ribosomal protein L18	C	No	yes	yes	13,349	123
STP_0065	A0A0F7XAU1	30S ribosomal protein S5	C	No	yes	yes	17,669	165
STP_0147	P73636.1	30S ribosomal protein S6	C	No	yes	yes	13,237	113
STP_0149	Q9A7Q2	30S ribosomal protein S18	C	No	yes	yes	10,114	92
STP_0303	Q6B906	50S ribosomal protein L1, chloroplastic	C	No	yes	yes	26,287	235

STP_0458	Q65VG0	50S ribosomal protein L19	C	No	yes	yes	13,072	116
STP_0542	A0A0E2Q2D4	50S ribosomal protein L35	E	No	yes	yes	7,810	66
STP_0555	Q88WN5.1	50S ribosomal protein L21	E, M	No	yes	yes	11,049	102
STP_0613	P21464	30S ribosomal protein S2	C	No	yes	yes	27,967	246
STP_0614	A0A089XR95	50S ribosomal protein L7/L12	C	No	yes	yes	12,408	121
STP_1663	Q889X5.1	30S ribosomal protein S7	C	No	yes	yes	17,666	156
STP_1769	O47115	Ribosomal protein subunit 2	C	No	yes	yes	22,934	204
STP_1819	Q8DWV3	50S ribosomal protein L32	E	No	yes	yes	6,922	60
STP_0141	Q6KIG7	Ribonuclease	M	No	yes	yes	26,239	227

STP_0519	Q9X4D0	DNA primase	C	No	yes	yes	67,111	597
STP_1841	Q65TC0	Replicative DNA helicase	C	No	yes	yes	54,367	485
STP_0140	Q9L3W2	Signal peptidase I	C	1	yes	yes	17,969	157
STP_0327	P30177	Uncharacterized protein YbiB	M	2	yes	yes	35,049	320
STP_0831	A0A0C6F VP2	Signal peptidase I	M	1	yes	yes	21,255	185
STP_1690	Q9KDP2	Membrane protein insertase YidC 2	M	5	yes	yes	32,108	280

Note: Seq. ID-Sequence ID provided by KEGG database for protein sequences; Protein ID-extracted from UniProt database proteins taxonomy& naming system; CELLO-database that provide information regarding cellular and sub-cellular localization of proteins; TMHMM-database for proteins' transmembrane helices; C-Cytoplasmic; M-Membrane; E-Extracellular; 3D structure information obtained from UniProt data base and ModBase.
